# Seasonal variation of child under nutrition in Malawi: is seasonal food availability an important factor? Findings from a national level cross-sectional study

**DOI:** 10.1186/1471-2458-14-1146

**Published:** 2014-11-05

**Authors:** Lana Clara Chikhungu, Nyovani Janet Madise

**Affiliations:** Division of Social Statistics and Demography, Centre for Global Health, Population, Poverty and Policy, Faculty of Social and Human Sciences, University of Southampton, Building 58, Social Sciences Academic Unit, Highfield, Southampton, SO17 1BJ UK

**Keywords:** Malawi, Stunting, Underweight, Seasonal variation, Child under nutrition, Child morbidity, Food availability

## Abstract

**Background:**

Child under nutrition is an underlying factor in millions of under-five child deaths and poor cognitive development worldwide. Whilst many studies have examined the levels and factors associated with child under nutrition in different settings, very little has been written on the variation of child under nutrition across seasons. This study explored seasonal food availability and child morbidity as influences of child nutritional status in Malawi.

**Methods:**

The study used the 2004 Malawi Integrated Household Survey data. Graphical analysis of the variation of child under nutrition, child morbidity and food availability across the 12 months of the year was undertaken to display seasonal patterns over the year. Multivariate analysis was used to explore the importance of season after controlling for well-established factors that are known to influence a child’s nutritional status.

**Results:**

A surprising finding is that children were less likely to be stunted and less likely to be underweight in the lean cropping season (September to February) compared to the post-harvest season (March to August). The odds ratio for stunting were 0.80 (0.72, 0.90) and the odds ratio for underweight were 0.77 (0.66, 0.90). The season when child under nutrition levels were high coincided with the period of high child morbidity in line with previous studies. Children that were ill in the two weeks prior to survey were more likely to be underweight compared to children that were not ill 1.18 (1.01, 1.38).

**Conclusion:**

In Malawi child nutritional status varies across seasons and follows a seasonal pattern of childhood illness but not that of household food availability.

## Background

Child under nutrition is one of the big challenges in global health. Estimates indicate that under nutrition is implicated in 50% of child deaths in developing countries every year and in the long-term contributes to poor cognitive development [1–3]. Whilst many studies have studied the levels and factors associated with child under nutrition in different settings in an attempt to identify better strategies to tackle the problem of child under nutrition, very little has been written on the variation of child under nutrition across seasons. In countries where the main source of food is own production like in Malawi, the dependence on rain fed agriculture creates variation in food availability across seasons. Periods after harvest are abundant with food whilst cropping periods have less food. It is no surprise that studies have established an association between the seasonal food variation and nutritional status amongst adults whereby seasonal food shortages have been associated with body weight losses of 2 to 6% of total body weight [4–7].

The few studies that have investigated the association between food availability and child nutritional status report inconsistent findings. In a study conducted in Ethiopia, children registered better weight for height z-scores in a period before harvest compared to a period after harvest whilst the pattern observed for adults was as expected, a higher average Body Mass Index was reported in the season of plenty and a lower average Body Mass Index in the lean season [8]. On the other hand, a Kenyan study did not find significant seasonal differences in children’s mean weight changes but the percentage of children stunted was higher during the lean season (51%) compared to the post-harvest months (28%) [9]. Other studies have compared children’s nutritional status between the wet season and the dry season and have found than children are more likely to have poorer nutritional status in the dry season compared to the wet season [10, 11]. Similarly, a study conducted in rural Malawi reports of significantly lower levels of wasting (3%) from August to December (a mixture of dry and wet months) compared to March a dry month (6%). The same study however found that there was a rapid decline in children’s weight for age z scores and height for age z scores for children aged 1 to 6 months and those aged 13 to 36 months in the rainy season compared to the dry season [12].

Some studies have reported an association between seasonal morbidity and child nutritional status suggesting that seasonal morbidity may be a bigger contributor to seasonal variation in child nutritional status than the seasonal variation in food availability [13–15]. A study of the nomadic Turkana children reports that infection may be an important contributor to the high levels of nutritional and immunological stress than food availability alone [16]. On the other hand seasonal variation in agricultural activities may also impact on the amount of time mothers allocate for child care. A theoretical framework of the link between the nutritional status of households and food security stipulates a cause and effect relationship between the two and specifies that the ways that agricultural changes may affect food consumption and nutrition include time allocation especially of women which in turn influences child care, food preparation and energy nutrient expenditures [17]. Mothers may reduce their time for childcare during and after harvest time [18–20] and this may contribute to poor nutritional status of children [19, 21]. The importance of childcare in child nutritional status is echoed by the findings in India that the relative risk of stunting and underweight for a child of a working mother was higher than that of a non-working mother after controlling for child’s age and the income of the household [22]. Furthermore, in the Bolivian Andes, heavy agricultural workloads and lack of support for child feeding from spouses and mother-in-laws were cited as barriers to improving infant and young child feeding practices [23].

Anecdotal evidence from Kasungu district in Malawi (where tobacco is the main cash crop) suggests that mothers may decide not to take their undernourished children to nutrition rehabilitation centres during the busy tobacco processing periods [24]. On the other hand, household income gains associated with cash crop production may not have an immediate or large improvement on pre-schooler nutritional status [25]. Previous studies in Malawi report that age of the child, child illness, the region of residence, rural or urban residence, and household socio-economic status are some of the important factors associated with a child’s nutritional status [26–29].

Whilst it is clear that households in Malawi experience variation in food availability across seasons, it is not known if at the national level, the variation of child under nutrition follows a pattern similar to that of food availability or that of child morbidity. Such information would be important in identifying the types of interventions that are required to tackle child stunting and underweight in Malawi, currently estimated at 47% and 13% respectively [30]. In this study, we analyse the variation of child under nutrition, child morbidity and household food availability across seasons in Malawi. We hypothesize that child under nutrition will vary between the seasons following the pattern of seasonal food availability, such that the lean period should have higher levels of undernourished children whilst the post-harvest period should have lower levels of undernourished children. Since the lean period is also the wet season when children are at increased risk of diarrhoea and malaria, we also hypothesize higher child morbidity and hence higher under nutrition in the lean, wet periods compared to the post-harvest, dry periods.

## Methods

We used the 2004 Malawi Integrated Household Survey (IHS 2) data which were collected from March 2004 to March 2005 by the National Statistical Office of Malawi. The (IHS 2) data were made available for use to the authors by the National Statistical Office of Malawi and are available for use by the general public through sending a request to the National Statistical Office of Malawi. The IHS 2 is similar to the World Bank sponsored Living Standards and Measurement Surveys and was a nationally representative cross-sectional study. The sample for IHS 2 was drawn using a two-stage stratified sampling procedure. The sample frame included all three regions of Malawi; north, centre and south. The country was stratified into urban and rural strata. The first stage involved selection of enumeration areas for each stratum on the basis of probability proportional to size based on the enumeration area listing from the 1998 Population and Housing Census. In the second stage, 20 households were randomly selected from each enumeration area, which resulted in a total of 11,280 households eligible for interviews. The households eligible for interviews were grouped into 12 groups in each district such that each group was interviewed once across the twelve months of the year. Interviews were conducted in 10,777 households through a single visit representing a 96% response rate. Interviews were conducted with household heads to collect household data, whilst mothers and guardians provided children’s information. In households with children aged 6 to 59 months, child anthropometric measurements were taken. In some cases households had to be visited twice if children were not available during the first visit. Anthropometric data was available for 6,687 children and this is the sample that was used in this study. More information relating to the survey design and how the data were collected can be accessed via the National Statistical Office of Malawi website: http://www.nsomalawi.mw/.

Among others, the IHS 2 data have information on household’s food and non-food expenditure in the past week, household poverty status^a^, illnesses in the past two weeks, participation in nutrition programmes and participation in under-five clinics which provide for rich analyses in relation to factors associated with child under nutrition. To ensure comparability of data on food expenditure across the year, z scores were obtained on expenditure on food separately for those interviewed in the period September to February (the lean months) and those interviewed from March to August (the post-harvest period) and then the data were merged together for analysis. Since the data were collected throughout the year across all districts, it is possible to estimate stunting, underweight, morbidity and household characteristics for Malawian children across different time periods of the year. There is missing information on the following variables: participation in nutrition interventions (0.13%), participation in under-five clinics (0.10%), quality of housing (24.5%) toilet facility (25.6%) and qualification of household head (25.6%). We maintained the missing cases on the variables on quality of housing, toilet facility and qualification of household head by including a category for the cases with missing data in each of the variables.

The height-for-age z-scores and the weight-for-age z-scores were computed using the WHO Anthro software version 3.2.2 based on the 2006 WHO growth standards [31]. Before importing the height data into Anthro software, heights greater than 150 cm or lower than 38 cm, which constituted less than 0.5% of the total sample, were set at missing due to implausibility [32]. The height for age z- scores were all within the range of -6 and 6 in line with the WHO recommendations on plausible range for height-for-age z scores. An investigation of the distribution of the height for age z scores (haz) and weight for age z scores (waz) indicated that both the haz and waz were not normally distributed and as such we computed binary response variables instead of treating the response variables as continuous. Based on the WHO recommendations, a child with a height-for-age z score of less than -2 is defined to be stunted and a child with a weight-for- age z score of less than -2 is defined to be underweight. We assigned a value of 1 if the child is stunted or underweight and 0 otherwise. Stunting is an indicator of chronic under nutrition while underweight is a composite measure of chronic (stunting) and acute under nutrition (wasting) [33].

In general, seasons in Malawi can be broken into four distinct periods: the warm and wet season from December to February with heavy rains during which cropping takes place; the warm and dry season from March to May; the cool and dry season from June to August and the hot and dry season from September to November. November however is variable since heavy rains can start in this month, particularly in the southern parts of the country. To simplify the weather terminologies the months from September to February are defined as the wet season whilst the months from March to August are defined as the dry season. To analyse food availability across the year, an exploration of annual household food expenditure^b^ across the year is done. In terms of seasonal food availability, the months from September to February are grouped together as they depict the lean months whilst the months of March to August are characterised by better food security following the harvests. On average all the months in the harvest period are in the dry season and all the months in the lean period belong to the wet season. It is therefore possible to analyse the levels of child morbidity across the weather seasons linked to the food availability seasons.

We undertook preliminary analyses using graphs to assess similarities/differences in the patterns of food availability, child under nutrition and child morbidity across the survey year. Chi- square tests were used to test the association between stunting or underweight and a range of child-level and household socio-economic characteristics. The choice of socio-economic variables used in this study was informed by published literature on the determinants of child nutritional status and child survival and findings from previous studies in Malawi. These variables are described in Table 1 [26–29, 34–38]. A P Value cut off point of 0.05 was used to establish if the association was statistically significant. We checked for multicollinearity between the predictor variables by assessing the correlation of their coefficients after running the regression and were satisfied that the correlation was low; r < -0.5 for all the coefficients. The highest correlation was between the coefficient of the missing category of the variable on improved floor and permanent roof and the coefficient of the missing category of the variable on qualification of the household head which was estimated at -0.47 in the stunting model and -0.49 in the underweight model.

**Table 1:** **Characteristics of the under-five children (6-59 months) studied, IHS-2 data**

Variable	Percentage	N
Period of interview is March to August	60.0	6,687
**Child characteristics**		
Stunted	50.1	6,687
Underweight	12.7	6,687
Participates in a nutrition intervention	3.5	6,678
Participates in an under-five clinic	60.6	6,680
Female	51.1	6,687
Children ill in the past two weeks	39.6	6,687
**Household characteristics**		
Household has a toilet facility	84.0	6,687
Household had piped or protected water source	66.9	6,687
House has no permanent roof	17.9	6,687
House does not have an improved floor	81.5	6,687
Household head has no education qualification	75.8	6,687
Household head has primary education	10.6	6,687
Household head has junior secondary school education or higher	14.4	6,687
Poor	55.2	6,687
**Location characteristics**		
Urban	9.6	6,687
North	11.9	6,687
Central	42.5	6,687
South	45.6	6,687

Previous studies undertaken in Malawi have reported clustering of underweight in households and communities [27, 39]. Our initial exploration of the data indicated that about 23% of the households had two children under the age of five and that the average number of children per community was 123 which entailed the need to undertake a multilevel model [40]. However, the stunting and underweight variances at both the household and community levels were no longer significant after including predictor variables and therefore we undertook a single level model. The fitness of the final model was tested by obtaining a classification table to identify the percentage of cases that the models correctly specified.

We used the logit link, a function that models the probability that a child is stunted or underweight as follows:


Where *x*_1_ to *x*_11_ represent explanatory variables that are described in Table 1, *β*_0_ is the overall intercept and to  are coefficients for the explanatory variables *x*_1_ to *x*_11_.

## Results

Table 1 presents a summary of socio-demographic and economic characteristics of the children studied. The estimate for underweight among children aged 6-59 months is 12.7% and the estimate for stunting is 50.1%. The table also shows that just over half (51.1%) of children were female, 39.6% were ill in the two weeks before the interview, only 3.5% attended a nutrition intervention, 55.2% of the households were poor, 81.5% did not have an improved floor and 75.8% of the household heads had no education qualification. The mean age of the studied children was 32 months.

### Factors associated with stunting and underweight

Table 2 presents the results of the Chi square test of the association between stunting and underweight and various characteristics analysed in this study.

**Table 2:** **Association between stunting, underweight and various characteristics, IHS-2 data**

Variable	% stunted	Chi square P Value	% underweight	Chi square P Value	N
Interviewed March to August	52.0	<0.001	13.8	<0.01	4,012
Interviewed September to February	47.2		11.0		2,675
Participates in a Nutrition Intervention	59.9	<0.01	25.1	<0.001	226
Does not	49.6		12.2		6,452
Participates in under-five clinic	49.8	0.59	13.9	<0.001	4,137
Does not	50.5		10.8		2,543
**Child characteristics**					
Male	52.5	<0.001	14.5	<0.001	3,279
Female	47.6		11.0		3,408
Child was ill	50.5	0.56	14.0	0.02	2,661
Child was not ill	49.8		11.9		4,026
**Household characteristics**					
Has a toilet facility	50.6	<0.001	12.1	0.09	4,216
No toilet	55.1		12.5		760
Missing	46.4		14.3		1,711
Has piped or protected water source	49.4	<0.001	10.9	<0.001	3,333
Neither piped nor protected water source	55.2		14.6		1,610
Missing	46.5		14.2		1,711
Has permanent roof and improved floor	41.8	<0.001	7.0	<0.001	812
Has either a permanent roof or an improved floor	49.9		9.1		485
No permanent roof and no improved floor	53.5		13.6		3,686
Missing	46.3		14.3		1,704
Household is poor	51.1	0.04	13.4	0.08	3,710
Household is not poor	48.3		11.8		2,977
Household head qualification					
No qualification	53.2	<0.001	13.3	<0.001	3,693
Primary school certificate	48.2		9.9		570
Junior secondary school or higher	43.2		7.7		715
Missing	46.4		14.2		1,709
**Location characteristics**					
Northern region	47.9	<0.001	11.7	0.09	1,093
Central region	53.2		13.7		2,645
Southern region	47.7		12.0		2,949
Urban	46.6	0.07	11.3	0.28	713
Rural	50.4		12.8		5,974

The results show that stunting and underweight levels are significantly higher amongst children from households that were interviewed between March and August, those participating in a nutrition intervention, male children, children from households without piped or protected water source, children from households without a permanent or an improved floor and children from households where the head had no qualification compared to their counterparts. Stunting is also significantly higher amongst children from households without a toilet facility, households identified as poor, those from the central region compared to their counterparts whilst underweight is higher amongst children that were participating in an under-five clinic and those that were ill compared to their counterparts.

### Variation of food availability by month of interview

**Figure 1:**
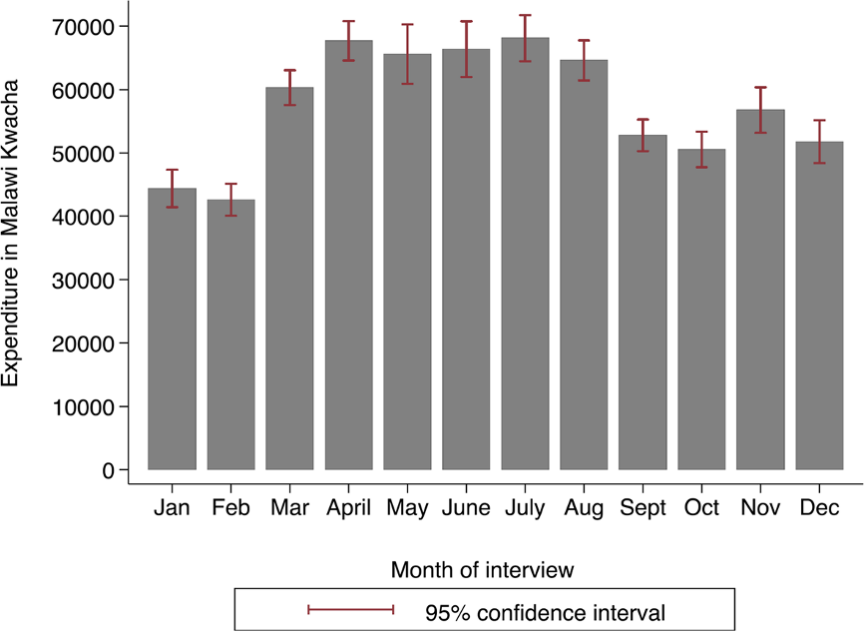
**Annual household food expenditure by month of interview (IHS 2 data).**

The results for the analysis of household annual food expenditure show that household annual food expenditure is significantly higher for the households that were interviewed in the months of March to August compared to households that were interviewed in the months of September to February as shown in Figure 1. A similar pattern was obtained for the analysis based on standardised annual household food expenditure.

### Variation in child under nutrition by month of interview

**Figure 2:**
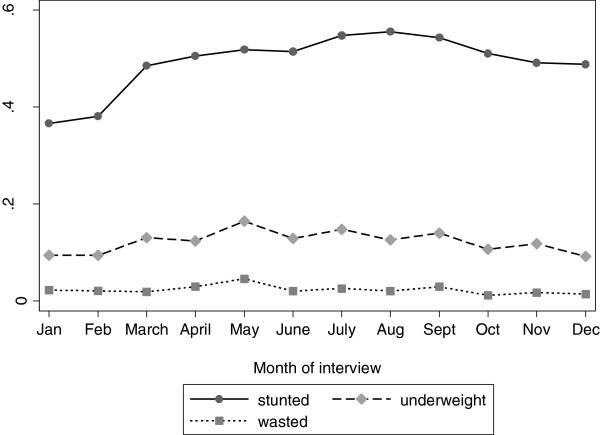
**Proportion of children stunted, underweight and wasted by month of interview (IHS 2 data).**

It is expected that child under nutrition should vary across the year since availability of food varies across the year and because the rate of child illnesses may differ across different seasons. In Malawi, periods soon after harvest which is around March/April have abundant food whilst from November to February there is severe shortage of food. This is supported by analysis shown in Figure 1. An analysis of the proportion of children stunted, underweight and wasted by month of interview is given in Figure 2. The Figure shows that the proportion of children stunted is much lower in January and February compared to all other months. On average, the proportion of children stunted is lower in the months of September to February compared to the months of March to August. A similar pattern is observed for the proportion of children underweight and the proportion of children wasted. The proportions of children stunted, underweight and wasted for households that were interviewed in the period of March to August were 0.52, 0.14 and 0.03 respectively whilst for the months of September to February these were 0.47, 0.11 and 0.02 respectively. The difference in the proportion of children stunted and those underweight was statistically significant (P value <0.01), but not significant for wasting (P value 0.07).

### Variation of child morbidity by month of interview and season

**Figure 3:**
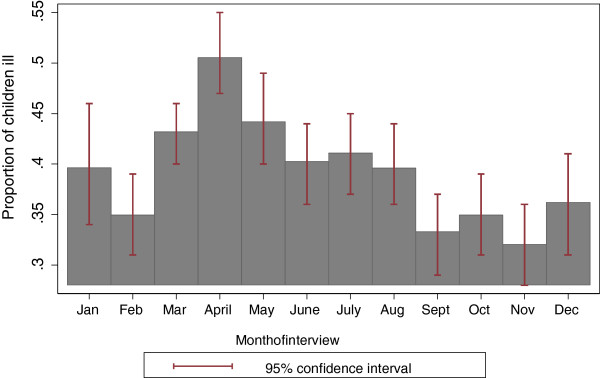
**Proportion of children ill by month of interview (IHS 2 data).**

Considering the important association between child morbidity and child under nutrition and the fact that child morbidity might vary across seasons as shown in previous studies, this section analyses how child illnesses in general vary across the interview months and the different seasons in Malawi. Figure 3 shows the proportion of children that were ill in the last two weeks for each month. Child illnesses included diarrhoea, lower respiratory infections, fever, stomachache and upper respiratory infections. The proportion of children who were ill is significantly higher in April compared to the rest of the months, although not significantly different from the proportion of children ill in the month of May. On average the estimate for the percentage of children ill in the months of March to August (the dry season) was 42.8%, and was higher than in the months of September to February (predominantly wet season), 34.8% P value <0.01. Based on the above analysis, the period of high child illness is similar to the period with high stunting and underweight rates shown in Figure 2. However, contrary to our hypothesis, the period of high child illness is the dry season and not the wet season.

### Multivariate analysis results

The bivariate analysis indicate that the levels of stunting and the levels of underweight are higher in the months of March to August compared to the months of September to February. To further confirm if the difference in stunting and underweight rates between the two periods is statistically significant, multivariate analysis were undertaken controlling for factors presented in Table 1. The classification table obtained after running the stunting and underweight regression models to check for model fitness indicated that 59.5% of cases were correctly specified in the stunting model whilst 87.5% cases were correctly specified in the underweight model. The multivariate analysis results are given in Table 3. The results show that the period of interview is significantly associated with a child’s stunting status and child’s underweight status. Children from households that were interviewed between September to February were less likely to be stunted and were less likely to be underweight compared to children from households that were interviewed in the months of March to August; odds ratio 0.80(0.72, 0.90) and 0.77 (0.66, 0.90) respectively. The odds of stunting were lower for female children compared to male children 0.80(0.72,0.89) and the odds of underweight were also lower for female children compared to male children 0.72(0.62,0.84). Children that were ill in the two weeks prior to the interview were more likely to be underweight compared to children that were not ill 1.18(1.01,1.38). The results appear to support our hypothesis that child under nutrition follows a seasonal pattern of child morbidity but not our hypothesis that child under nutrition follows a seasonal pattern similar to that of food availability. There was no significant association between a child’s chronic under nutrition, measured by stunting status, and child illness.

**Table 3:** **Multivariate analysis results for children’s stunting and underweight, IHS-2**

Variable	Stunting odds ratio	95% confidence interval	Underweight odds ratio	95% confidence interval
Period of interview is September to February	0.80	(0.72,0.90)	0.77	(0.66,0.90)
Reference : March to August				
**Child characteristics**				
Female	0.80	(0.72,0.89)	0.72	(0.62,0.84)
Reference is Male				
Age squared	0.69	(0.65,0.73)	0.92	(0.84,1.01)
Age	1.42	(1.34,1.51)	1.13	(1.03,1.23)
Child was ill	1.10	(0.99,1.23)	1.18	(1.01,1.38)
Reference: Child was not ill				
**Household characteristics**				
Annual household expenditure on food	0.92	(0.87,0.98)	0.97	(0.89,1.06)
Medium housing quality ( Household has a permanent roof or an improved floor)	0.91	(0.74,1.12)	0.65	(0.46,0.93)
High housing quality (Household has both a permanent roof and improved floor)	0.70	(0.58,0.86)	0.53	(0.37,0.75)
Missing	0.78	(0.32,1.86)	1.71	(0.66,4.41)
Reference: Poor Housing quality (household has neither a permanent roof nor an improved floor)				
Household has a toilet facility	0.94	(0.80,1.11)	1.14	(0.89,1.46)
Missing	1.01	(0.46,2.18)	1.40	(0.55,3.55)
Reference: No toilet facility				
Household has protected water source	0.88	(0.77,1.00)	0.80	(0.66,0.97)
Missing	1.21	(0.69,2.11)	0.89	(0.35,2.24)
Reference: No protected water source				
Household head’s qualification:				
Primary school certificate	0.89	(0.73,1.06)	0.78	(0.57,1.06)
Secondary school certificate or higher	0.84	(0.70,1.03)	0.71	(0.50,1.00)
Missing	1.00	(0.43,2.36)	0.51	(0.19,1.35)
Reference: No education)				
**Location characteristics**				
Central region	1.19	(1.03,1.39)	1.13	(0.90,1.42)
Southern region	0.96	(0.82,1.12)	1.03	(0.81,1.30)
Reference: Northern Region				
Rural	0.93	(0.77,1.13)	0.81	(0.60,1.07)
Reference: Urban				

The quality of housing (whether a household has a permanent roof or an improved floor or both) had a significant association with both stunting and underweight status. This factor appeared to be particularly important to a child’s underweight status compared to a child’s stunting status. Compared to children from households that had neither an improved floor nor a permanent roof, the odds of underweight were 0.53(0.37,0.75) and the odds of stunting were 0.70(0.58,0.86) amongst children from households that had both a permanent roof and an improved floor. The annual household food expenditure was only significantly associated with a child’s stunting status. A one unit increase in the household’s mean z score of food expenditure was associated with a reduction in the odds of stunting by 8%. Children from households with access to piped water or a protected water source had lower odds of underweight compared to children from households without access to piped or protected water source; 0.80 (0.66,0.97). The odds of stunting were significantly higher for children from the Central region compared to children from Northern region; 1.19(1.03,1.39). No association was established between a child’s nutritional status and factors such as toilet facility and residing in urban or rural areas.

## Discussion

This study has found that child under nutrition varies across seasons in Malawi. The months of March to August which comprise the harvest season and also the dry weather season have significantly higher levels of stunting and underweight compared to the months of September to February which comprise the lean cropping months and the wet season. Children are more likely to be ill during the months of March to August compared to the months of September to February and children that were ill in the two weeks prior to the survey were more likely to be underweight. There is a significant association between annual household expenditure on food and a child’s stunting status. Although the months of March to August which have better access to food have higher levels of child under nutrition, the multivariate results indicate that household annual expenditure on food overall is positively associated with better children’s nutritional status, in line with previous studies that indicate the importance of household socio-economic status in children’s nutritional status [28, 34, 35, 41]. This suggests that the explanations for the finding from this study of higher stunting and underweight levels amongst children from households that were interviewed in the harvest season (March to August) compared to those from households that were interviewed in the hunger season (September to February) may not be wholly to do with food availability.

Based on anecdotal evidence [42], the pattern of undernourished children requiring admissions in Malawi does follow the trend of food scarcity shown in Figure 1 with more admissions occurring during the months of January to February. This pattern appeared to be supported by statistics from the Nutrition Unit of the Malawi Government’s Ministry of Health on admissions for severely undernourished children which also showed that the highest number of children on the Supplementary Feeding Programme (SFP) and the Outpatient Therapeutic Programme (OTP) in 2011 was in the months of January and February [43]. The fact that in the hunger months more undernourished children are treated, as shown by the higher levels of OTP admissions and that more children are on the SFP compared to the harvest time could also explain the lower stunting and underweight rates experienced during this period, since those severely undernourished may have been in hospitals when the household interviews were conducted, whilst on the other hand the SFP could be contributing to the relatively better nutritional status of children during the lean months. Although the criterion for the OTP is acute malnutrition, particularly wasting, it is possible that some of the children that are wasted may also be stunted or underweight. Not only does evidence suggest that wasting is closely associated with stunting [44, 45] but stunted children may have physical indicators of malnutrition which reveal poor short-term health growth such as a lower mid arm circumference, skin discoloration and thin limbs [46].

Whilst the unexpected relationship between food availability and child nutritional status could be explained by there being a lag between a child suffering from hunger and being affected by under nutrition, which would be particularly true for stunting which measures chronic under nutrition, the findings of this study that the other two common measures of child under nutrition, wasting (which measures short term under nutrition) and underweight (a composite of short term and long term under nutrition) followed a pattern similar to that of stunting do not support such explanation.

Although these findings are not what one would expect, they are in line with studies that have reported poorer children’s nutritional status in the dry months compared to the wet months since in Malawi the harvest season is dry and the lean season is wet [10, 11]. It is possible that although households have less maize (Malawi staple food) and other foods that are produced through farming during the months of September to February, households may have abundant food that naturally grow with the rains i.e. mangoes, pumpkins and other vegetables which are rich in vitamins i.e. vitamin A, and C that are also vital for child health and nutritional status [47–49]. But still, there could be other factors that also vary across seasons that influence children’s nutritional status. Seasonal food availability could not explain seasonal variation in growth velocity amongst Shanghai infants and seasonal changes in child nutritional status in Ethiopia [8, 50].

Another likely explanation of the unexpected relationship between food availability and child stunting status established in this study could be that although food availability varies across the year, food consumed by children does not really vary across the year to an extent of influencing a child’s nutritional status, and that perhaps other factors such as child illness that also vary across the year are more influential than food availability. This explanation appears to be supported by the pattern shown in Figure 3 which shows that on average child illnesses are higher in the months of March to August. Although child diarrhoea could not explain the seasonal variation in growth velocity amongst Shanghai infants, the role of infection and illness across seasons and its association with child nutritional status has been reported in several settings [8, 14, 15, 50]. Conceptual frameworks on child nutritional status cite child morbidity as one of the factors that is very closely associated with child nutritional status, whilst household food availability may influence a child’s nutritional status through a child’s nutrient intake [36, 37]. Child morbidity may have a greater impact on a child’s nutritional status compared to nutrient intake per say by reducing a child’s uptake of nutrients through loss of appetite, increased metabolism and reduced absorption of nutrients. Prolonged morbidity may result in children getting caught up in a vicious circle of poor health and poor nutrition, thus jeopardising their chances of recovering from any illness quickly [51–53]. The role of childhood illnesses in influencing a child’s nutritional status in general has been widely reported [27, 39, 53, 54].

It is also possible that parents and guardians whose main livelihood is farming reduce the amount of time allocated for child care during the busy harvesting times [18, 19]. One way that agricultural changes may affect food consumption and nutrition is time allocation [17]. The reduced time allocated for child care could be a contributing factor to the lower numbers of children participating in nutrition interventions during the harvest time in Malawi especially amongst communities whose main livelihood is tobacco farming such as in Kasungu [24]. Whilst household wealth plays an important role in influencing better nutritional status for children [35, 41] the benefits of income obtained through cash crop production to children’s nutritional status may not be immediate or large [25] and may be outweighed by the less time that women have to spend for child care [19, 21–23].

The estimate for underweight of 12.7% in this study is much lower than that from the 2004 Malawi Demographic and Health Survey report at 18.5% [55]. This is probably because different types of weigh scales were used in taking weight measurements for children. The IHS 2 used salter scales whilst the 2004 MDHS used electronic scales. It is possible that the underweight estimate from the IHS 2 is underestimated due to a higher likelihood of approximation in salter scales. The estimate for stunting among children 6-59 months of age from IHS 2 is also lower estimated at 50.1% compared to 52.7% from the 2004 MDHS, but the difference is not huge. The likely effect of the difference in the estimates is that, the power to detect significant factors may be reduced. However, this may not have a large impact on our study findings whose main focus was to explore seasonal variation of child under nutrition and the role of food availability and child morbidity. Since our study is based on cross-sectional data, we are not able to establish any causation between factors at different time points but are only able to describe differences in child nutritional status in different seasons and the potential explanatory factors.

## Conclusions

Child under nutrition in Malawi varies across seasons and the pattern is similar to that of the seasonal variation in child illnesses. On average, periods with a higher proportion of children that are affected by illnesses have a higher proportion of children that are undernourished and child illness is a significant predictor of underweight. Since the harvesting periods are dry whilst the lean period is wet, the lower levels of child under nutrition during the lean, wet periods could be explained by better access to nutrient rich foods that grow with the rains. On the other hand reduced child care during the busy harvesting period could also contribute to the poor child nutritional status experienced during this period. The Malawi Government should therefore intensify its community outreach component of the Community-based Therapeutic Care for the management of acute malnutrition but also consider implementing child health promotion programmes during the months of March to August, the period when child morbidity is high.

## Endnotes

^a^Poor households are those below the national poverty line. The poverty line is the minimum expenditure required to meet the minimum daily calorific requirements plus some minimum non-food expenditure.

^b^Annual household food expenditure is the monetary value of what the household consumed in the past 7 days before the day of interview in Malawi Kwacha multiplied by 52 (weeks) and is adjusted for spatial and temporal price deflators. It includes consumption from own production, purchases, as well as gifts and other sources.

## Authors’ information

LC (PhD) is a Teaching fellow at the University of Southampton.

NM (PhD) is Professor of Demography and Social Statistics at the University of Southampton.
